# Editorial: Deciphering transcriptional dynamics in plant responses to viruses

**DOI:** 10.3389/fpls.2023.1153747

**Published:** 2023-02-10

**Authors:** Venura Herath, Jeanmarie Verchot

**Affiliations:** ^1^ Department of Agriculture Biology, Faculty of Agriculture, University of Peradeniya, Peradeniya, Sri Lanka; ^2^ Department of Plant Pathology & Microbiology, Texas A&M University, College Station, TX, United States

**Keywords:** viruses, cistrome, transcriptional regulatory network, plant immunity genes, plant virus interaction

The global burden of plant viruses is significantly affecting the world’s food production. The impact is more localized to the regions where there is a very high population density with limited access to resources. This situation is further worsened by the impact of global climate change (Savary et al., 2019). Combatting plant viral infections is considered one of the top priorities by plant breeders around the globe. Until recently most of the breeding efforts were restricted to mass screening of cultivars and incorporating virus-resistant traits into new cultivars and controlling the insect vectors using various pesticides. Conventional breeding approaches can take years to produce a commercially desirable crop variety while the same extended time creates an unfavorable situation given the viruses’ ability to rapidly evolve and overcome host genetic resistances. With the rapid advancement of next-generation sequencing technologies now both basic and applied plant researchers have the opportunity to explore the plant host responses and influence of both intrinsic and extrinsic factors at the transcriptomic level at a global scale (Zanardo et al., 2019). This greatly reduces the time required to decode the transcriptional regulatory networks involved. As a result, scientists can identify the major signal transduction hubs and manipulate their expression levels spatiotemporally to make virus-resistant plants.

Xie et al. are looking at wheat streak mosaic virus (WSMV); one of the major diseases that affect wheat production globally. They were able to confirm that the Wsm2 locus is the major locus that controls the WSMV resistance in wheat. Then, they looked at the genetic variations in the locus and came up with potential candidate genes that can be used in future breeding programs.

The distribution and frequency of *cis-*regulatory elements on gene promoters can determine the spatiotemporal gene expression against virus attacks. Ke et al. are doing a deep dive into the regulation of *Nicotiana bentamina* argonautes 5(*NbAGO5*); a major gene that is involved in plant defense responses. They highlight the role of NbNAC42 and NbZF3 in the fine regulation of NbAGO5 mediated *via* the *NbAGO5* promoter.

External environmental factors can greatly influence plant responses to viruses. Boron is known to enhance plant defense against viruses (Buoso et al.). Guo et al. show that the boron-induced reactive oxygen species (ROS) signaling pathways are responsible for the Cucumber green mottle mosaic virus (CGMMV) resistance using *N. benthamina* and watermelon plant systems. Their results have a wide application in the fields where farmers can achieve virus resistance by modifying their agronomic practices.

Tan et al. demonstrate the functional characterization of UDP-Glycosyltransferases which are known for their antiviral properties. Their findings lay the groundwork for mass production of the UDP-Glycosyltransferases using novel synthetic biology approaches.

## Author contributions

VH wrote the article; VH and JV have revised the manuscript. All authors contributed to the article and approved the submitted version.
